# Supplementary material for: Identification of photoperiod-induced specific miRNAs in the adrenal glands of Sunite sheep (Ovis aries)
Source: Front Vet Sci. 2022 Jul 22;9:888207. doi: 10.3389/fvets.2022.888207 (PMC9354845; doi:10.3389/fvets.2022.888207)
Supplement: Supplementary file 1 [file Data_Sheet_1.ZIP › Supplementary file/miRDeep2 file.zip!/miRDeep2 file/Novel_Structure/LP42A2/Novel_19.pdf]

Provisional ID	:	NC_019464.2_15578
Score total	:	34.3
Score for star read(s)	:	3.9
Score for read counts	:	27.1
Score for mfe	:	1.7
Score for randfold	:	1.6
Score for cons. seed	:	
Total read count	:	65
Mature read count	:	64
Loop read count	:	0
Star read count	:	1

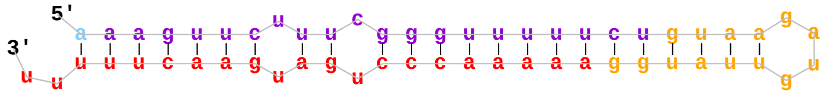

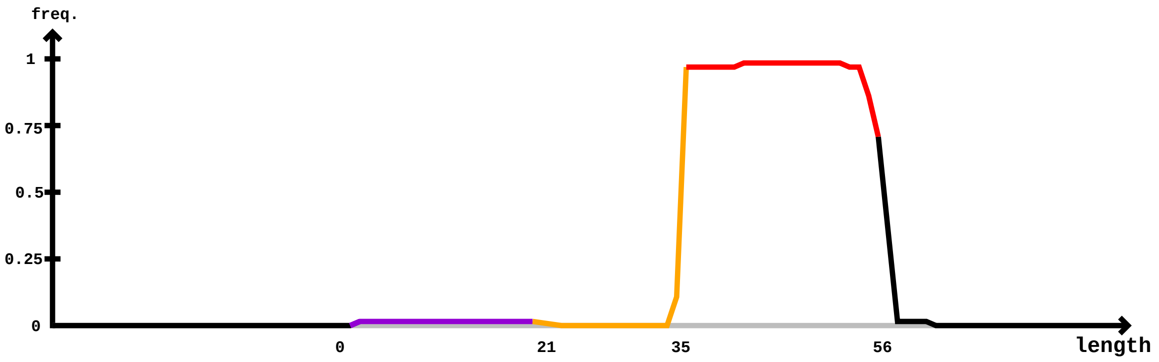

Star **Mature**

```
5'- cuuugcaugcuuucuauugaguugaccagaaaguucuuucggguuuuucuguaagauguuaugggaaaaacccugaugaacuuuuuggccaaccuuuccaacagcccuaau   -3'    obs
    cuuugcaugcuuucuauugaguugaccagaaaguucuuucggguuuuucuguaagauguuaugggaaaaacccugaugaacuuuuuggccaaccuuuccaacagcccuaau          exp
    ........(((.........((((.(((((((((((.((.((((((((((((((....)))))))))))))).)).))))))))))).)))).........)))......   reads     mm        sample
    ..............................aaguucAuucggguuuuucu............................................................   1         1         seq
    ...............................................................gaaaaacccAgaugaacuuuu..........................   6         1         seq
    ...............................................................gaaaaacccAgaugaacuuuuu.........................   1         1         seq
    ................................................................aaaaacccAgaugaacu.............................   1         1         seq
    ................................................................aaaaacccAgaugaacuuu...........................   7         1         seq
    ................................................................aaaaacccAgaugaacuuuu..........................   4         1         seq
    ................................................................aaaaacccAgaugaacuuuuu.........................   42        1         seq
    ................................................................aaaaacccAgaugaacuuuuug........................   2         1         seq
    ......................................................................ccugaugaacuuuuuggcca....................   1         0         seq
```
